# Expression, Purification, and Characterisation of Recombinant Alginate Lyase (*Flammeovirga* AL2) for the Bioconversion of Alginate into Alginate Oligosaccharides

**DOI:** 10.3390/molecules29235578

**Published:** 2024-11-26

**Authors:** Coleen E. Grobler, Blessing Mabate, Alaric Prins, Marilize Le Roes-Hill, Brett I. Pletschke

**Affiliations:** 1 Enzyme Science Programme (ESP), Department of Biochemistry, Microbiology and Bioinformatics, Rhodes University, Makhanda 6140, South Africa; g20g1213@campus.ru.ac.za (C.E.G.); bmabate@gmail.com (B.M.); 2 Applied Microbial and Health Biotechnology Institute, Cape Peninsula University of Technology, Bellville 7535, South Africa; prinsal@cput.ac.za (A.P.); leroesm@cput.ac.za (M.L.R.-H.)

**Keywords:** alginate, alginate lyase, alginate oligosaccharides, brown seaweeds, enzymatic hydrolysis

## Abstract

Alginate, a polysaccharide found in brown seaweeds, has regularly gained attention for its potential use as a source of bioactive compounds. However, it is structurally complex with a high molecular weight, limiting its application. Alginate oligosaccharides (AOS) are small, soluble fragments, making them more bioavailable. Alginate hydrolysis by enzymes is the preferred method for AOS production. Commercially available alginate lyases are limited, expensive, and sometimes exhibit unsatisfactory activity, making the search for novel alginate lyases with improved activity indispensable. The aims of this study were to codon-optimise, synthesise, express, purify, and characterise a recombinant alginate lyase, AL2, from *Flammeovirga* sp. strain MY04 and to compare it to a commercial alginate lyase. Expression was successfully performed using *Escherichia coli* ArcticExpress (DE3) RP cells, and the protein was purified through affinity chromatography. The recombinant enzyme was characterised by pH optimum studies, and temperature optimum and stability experiments. The optimal reaction conditions for AL2 were pH 9.0 and 37 °C, while for the commercial enzyme, the optimal conditions were pH 8.0 and 37 °C. At optimal reaction conditions, the specific activity of AL2 was 151.6 ± 12.8 µmol h^−1^ mg^−1^ protein and 96.9 ± 13.1 µmol h^−1^ mg^−1^ protein for the commercial alginate lyase. Moreover, AL2 displayed impressive activity in breaking down alginate into AOS. Hence, AL2 shows potential for use as an industrial enzyme for the hydrolysis of alginate into alginate oligosaccharides. Additional studies should be carried out to further characterise this enzyme, improve its purity, and optimise its activity.

## 1. Introduction

Brown algae (*Phaeophyta*) is one of the three main classes of macroalgae, commonly referred to as seaweeds [1]. The primary cell wall polysaccharides in brown seaweeds are fucoidans and alginates [2], with alginate being the predominant glycan. Brown kelp, a type of brown macroalgae, is a major source of polysaccharides and has shown potential in the agricultural, cosmetic, pharmaceutical, food, and biofuel industries [1]. Overall, these seaweeds are an attractive natural resource for producing bioactive compounds.

Alginate is an acidic, linear copolymer made up of two uronic acids, β-d-mannuronic acid (M) and its C5 epimer, α-l-guluronic acid (G) [2]. These hexuronic acid residues are randomly linked by α- and β-1→4 glycosidic bonds in varying configurations of homopolymers, i.e., MM and GG, and heteropolymers, i.e., MG blocks [3]. The β-1,4-glycosidic bonds link mannuronic residues, while guluronic residues are linked by α-1,4-glycosidic bonds [4]. The M/G ratios and polymer lengths differ between algal species, geographical locations, and the oceanic zone. The differences in uronic acid composition determine the molecular weights of the alginates, which range between 50 and 100,000 kDa [5].

Alginates are typically used in the food and medical industries due to their useful hydrophilic, chelation, and gelation properties [3,6]. Additionally, the bioactivities of alginates have recently become an important topic of research; however, the physical properties of alginates are limiting factors for their widespread application. Their high molecular weight, low solubility, and structural complexity greatly hinder the bioactivity and bioavailability of this polysaccharide [3,7].

Alginate oligosaccharides (AOS), the hydrolysis products of alginate, in contrast to alginates, have shown great potential for use as biopharmaceuticals due to their low toxicity, small size, non-immunogenicity, and efficient metabolism by the human body [3]. These molecules have been reported to display anti-diabetic activity, including insulin secretion in vitro and reducing the concentration of lipids and glucose from the bloodstream [4,8]. Alginate oligosaccharides have a degree of polymerisation of between 2 and 25 uronic acids and have been found to have various applications within a range of industries [3]. These oligos comprise the same three blocks as alginate (MM, GG, and MG). Their G/M ratio and size depend on algal species and the alginate lyase catalysing the depolymerisation reaction [3,9]. The bioavailability and bioactivity properties of AOS, compared to alginate, are more satisfactory in many cases due to their high solubility and lower molecular mass [9], allowing them to interact efficiently with biomolecules within the body.

Marine microorganisms associated with brown algae, including bacteria, fungi, and microalgae, cannot utilise alginate in its polymeric form because it is structurally complex; therefore, they naturally produce alginate lyases, which are hydrolytic enzymes, to digest this compound into a usable form [9]. These enzymes cleave the 1→4 glycosidic bonds that make up alginate to produce AOS. These lyases can be classified according to their substrate preference and cleavage patterns. Polymannuronate (M), polyguluronate (G), and polyMG-specific lyases are specific to MM, GG, and MG branches, respectively [9]. Alginate lyases can be further classified into exotype, endotype, and bifunctional lyases. Additionally, these enzymes can be characterised according to their molecular weights, being small (25–30 kDa), medium (~40 kDa), and large (>60 kDa) in size [9].

Alginate can be hydrolysed to produce AOS by several mechanisms, including chemical, acid and alkali, mechanical, physical, and enzymatic hydrolysis [3,10,11]. Enzymatic degradation is often the preferred method as it is highly specific—alginate lyases hydrolyse glycosidic bonds at fixed sites along the polysaccharide branch [2]. The reaction conditions are important to consider for different alginate lyases. Since these enzymes are typically isolated from a marine environment, they usually prefer more alkaline conditions and moderate temperatures, and are often dependent on the presence of sodium chloride [12].

Commercially available alginate lyases display low activity, and their physical properties limit their catalytic efficiency [1,10]. These enzymes are also very expensive, making alginate hydrolysis on an industrial scale a very costly process [10]. Recombinant alginate lyases have been produced to improve enzymatic activity and physical and chemical properties [1] for better performance during AOS production at an industrial scale. Although many alginate lyases have been isolated and characterised in terms of their physical and chemical properties, only some studies have investigated their enzymatic potential [8]. Therefore, it is essential to produce, purify, characterise, and investigate their efficiency during alginate hydrolysis, since AOS display potential as bioactive agents.

**Figure 1:**
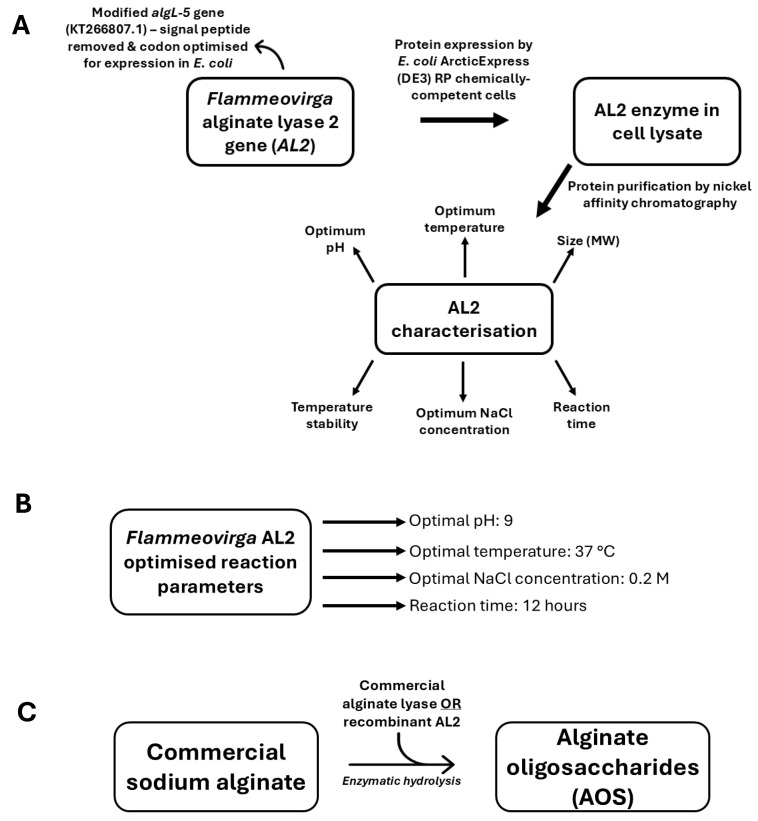
Flow chart summarising the workflow of this paper: (**A**) an outline of the expression, purification, and characterisation of *Flammeovirga* alginate lyase 2 (AL2); (**B**) optimised reaction parameters of the recombinant enzyme; and (**C**) a brief outline of the substrates and products involved in the hydrolysis reaction.

In this study, a truncated, codon-optimised alginate lyase from *Flammeovirga* sp. strain MY04 was recombinantly expressed, purified, and characterised to evaluate its potential as an alternative to the commercially available alginate lyase for producing AOS (Figure 1).

## 2. Results and Discussion

### 2.1. Expression of Recombinant AL2

**Figure 2:**
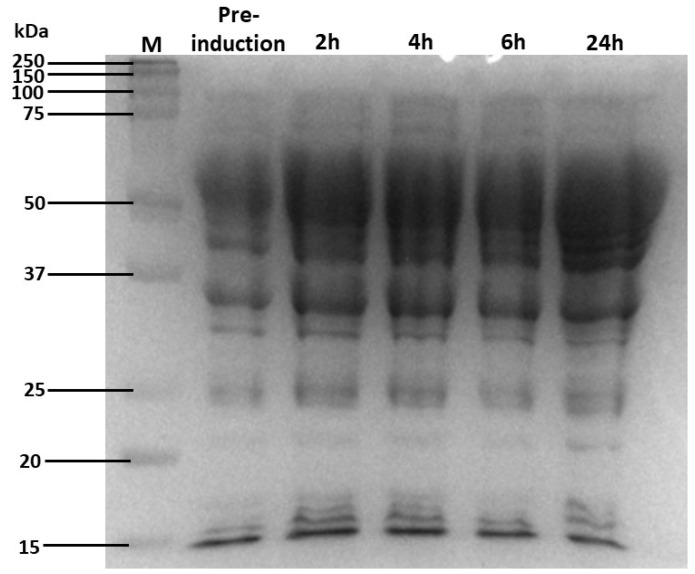
Expression of *Flammeovirga* alginate lyase 2 (AL2) in *E. coli* ArcticExpress bacterial cells using 12% sodium dodecyl sulfate-polyacrylamide gel (SDS-PAGE) stained with Coomassie Blue staining solution. Lane M—protein ladder (Precision Plus Protein^TM^ Standards, Bio-Rad, Hercules, CA, USA), lane Pre-induction: sample taken before IPTG induction, lanes 2 h, 4 h, 6 h, and 24 h: samples taken 2, 4, 6, and 24 h post-1 mM IPTG induction, respectively.

To confirm that AL2 was successfully expressed over 24 h, samples were collected before and after isopropyl β-D-1-thiogalactopyranoside (IPTG) induction, during which IPTG bound to the lac repressor, allowing for gene transcription. The samples were then analysed as shown in Figure 2. The recombinant protein, AL2, was successfully expressed by *E. coli* ArcticExpress (DE3) RP cells (Figure 2). Figure 2 shows that induction with IPTG dramatically increases protein concentration only 2 h after induction. Therefore, the induction step is necessary.

The protein concentrations remained relatively constant between 2 and 6 h, but at 24 h, much more protein was expressed, and a 24 h incubation period was therefore used for subsequent studies. The RP bacterial strain has been engineered for induction at low temperatures (temperatures lower than 20 °C), enhancing protein solubility. Therefore, leaky expression is possibly due to expression being carried out at a higher temperature (30 °C), decreasing protein solubility. Many studies have also reported optimal alginate lyase expression by *E. coli* hosts over 24 h at 30 °C [13,14,15]. Although the protein expression of AL2 was very similar at 37 °C, less energy is required to maintain a temperature of 30 °C in an industrial setting.

As seen in Figure 2, the protein was expressed at a relatively high concentration. The high expression levels were likely due to the removal of the signal peptide, which resulted in increased protein yields. The AL2 gene was also codon-optimised for expression in *E. coli*, allowing for expression at relatively high concentrations despite being a non-native enzyme in this bacterial species.

### 2.2. Purification of Recombinant AL2

**Figure 3:**
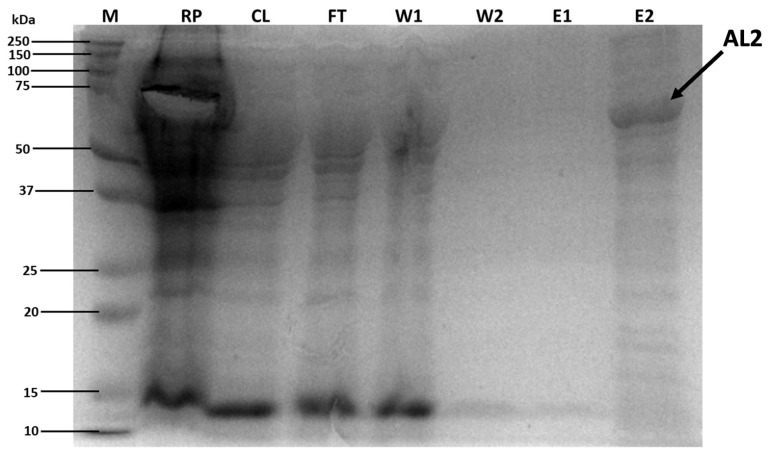
Protein purification of *Flammeovirga* alginate lyase 2 (AL2) from expression vector PET-28a (+) in *E. coli* ArcticExpress bacterial cells. SDS-polyacrylamide gel (12%) stained with Coomassie Blue staining solution. Lane M—protein ladder, lane RP—resuspended cell pellet (insoluble fraction), lane CL—cleared lysate (soluble fraction), lane FT—flow through, lanes W1 and W2—washes 1, and 2, respectively, lanes E1 and E2—elutions 1, and 2, respectively (partially purified AL2).

The samples collected during nickel-affinity chromatography purification were analysed on a 12% SDS-polyacrylamide gel to assess the purity of the recombinant enzyme after elution (Figure 3). As shown in Figure 3, a single, dominant, clear band, which is indicated by the arrow designated AL2, confirmed that the recombinant protein was partially purified by nickel affinity chromatography.

According to the protein ladder, the protein was ~60 kDa, as it migrated somewhere between 50 and 75 kDa (Figure 3), corresponding to the predicted size of 58.13 kDa after removal of the signal peptide. The truncated enzyme is, therefore, considered a medium-large alginate lyase, as enzymes >60 kDa are considered large alginate lyases [9]. Several characterised alginate lyases in the literature are very similar in size, ranging between 59 and 63 kDa [14,16,17].

The resuspended pellet (RP), containing the insoluble protein fraction, contained a high protein concentration, although a substantial amount of protein was present in the cleared lysate (CL) (Figure 3). The high protein concentrations in the RP could be attributed to the high level of expression that could overwhelm the cellular folding machinery, causing the recombinant protein to be insoluble. The insoluble protein fraction can typically be caused by inclusion bodies, which can form under suboptimal conditions such as above-optimal expression temperatures. Inclusion bodies form when proteins aggregate and are removed from suspension [18]. Although the bulk of the protein was trapped in the RP, the cleared lysate was selected for further purification as native purification is technically easier than denaturing and renaturing strategies like urea buffers. Furthermore, the cleared lysate (supernatant) had a substantial amount of protein for purification. A relatively large amount of protein was lost during the flow-through (FT) step, which showed that some recombinant proteins and the bacterial proteins did not bind to the nickel resin. During the first wash, some of our target protein, which was loosely bound, was washed off the column together with the native bacterially expressed proteins (Figure 3; lane W1). However, in the second wash (W2), the bound recombinant protein remained on the column, and no bacterial proteins were observed, meaning that all were washed off during the first wash (W1). We also observed that the protein could not be eluted using elution 1 buffer with 200 mM imidazole. However, elution 2 buffer with a high (500 mM) imidazole sample eluted a relatively clean and concentrated AL2 (Figure 3; lane E2), indicating a successful purification. However, small additional faint bands could be observed in the elution 2 (E2) sample, indicating protein degradation.

### 2.3. Alginate Lyase Characterisation

#### 2.3.1. Biochemical Characterisation

**Figure 4:**
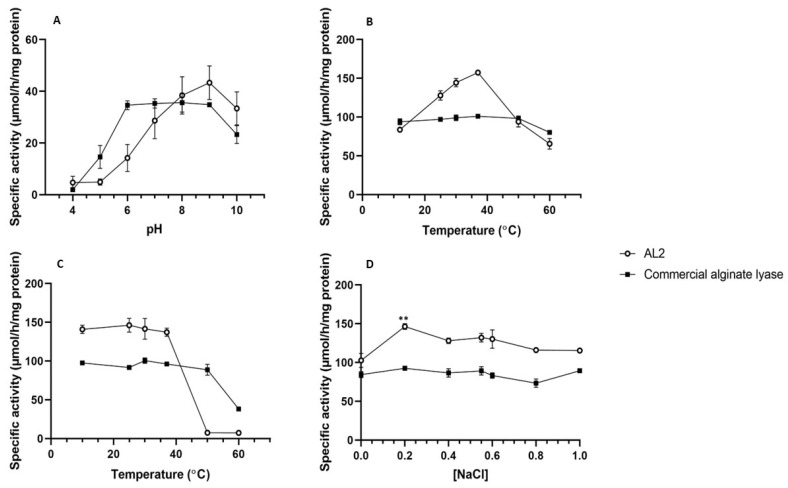
Biochemical characterisation of *Flammeovirga* sp. AL2 and a commercial alginate lyase regarding their optimum pH (**A**), temperature (**B**), thermal stability (**C**), and effect of NaCl concentration (**D**). Data points represent mean values ± SD (n = 3). ** denotes significant differences (*p* < 0.05) from the 0 M NaCl sample.

The enzymes were characterised by determining their optimum pH and temperature, as well as the effect of sodium chloride concentrations. Figure 4 summarises the optimal reaction conditions for the recombinant AL2 and commercial alginate lyase (Cat no. A1603, purchased from Sigma-Aldrich (St. Louis, MO, USA)).

The optimal pH for the commercial alginate lyase and AL2 was assessed by exposing the enzymes to a pH range of 4–10. The optimal pH of AL2 was pH 9, while the optimal pH for the commercial alginate lyase was pH 8 (Figure 4A). The commercial alginate lyase’s activity remained relatively unchanged between pH 6 and 9 (Figure 4A). These results agreed with other reports in the literature, as, according to [12], most characterised alginate lyases have an optimum pH of between 7 and 8.5. This is expected, as seawater has an average pH of 8.1, although it can range between pH 7.4 and 8.6 [19]. Similarly to the commercial alginate lyase, several alginate lyases displayed an optimum pH of 8, namely, AlgMsp from *Microbulbifer* sp. [20], A1-II from *Sphingomonas* sp. [21], and Algb from a *Vibrio* sp. [12]. AL2 had a high alkaline pH optimum, which was above the pH range of most other alginate lyases. However, it is not unique, as an alginate lyase from *Agarivorans* sp., a deep-sea bacterium, has been reported to have a pH optimum of 9.0 in the presence of NaCl and pH 10.0 in the absence of NaCl [15]. Interestingly, the enzyme from which AL2 is derived exhibited an optimal pH of 6.0 [22], indicating that the truncation and codon-optimisation affected the folding and activity of AL2.

The optimal temperature for the AL2 and the commercial alginate lyase was determined by incubating the enzymes over a temperature range of 12–60 °C. Both enzymes exhibited maximal activity at an optimal temperature of 37 °C (Figure 4B). Figure 4B also indicates that the activity of the commercial alginate lyase remained relatively consistent between 12 and 50 °C, with only a slight decrease at 60 °C. In contrast, a rapid decline in activity from 37 °C to 60 °C was observed for AL2. The commercial alginate lyase and AL2 are mesophilic enzymes that display the highest activity at moderate temperatures (20–45 °C). Many other alginate lyases display temperature optima within 30–40 °C [12]. *Flammeovirga* rAly5, from which AL2 is derived, had an optimal temperature of 40 °C, and, similarly to AL2, showed a rapid decline in activity at temperatures of 50 °C and above [22]. AlgA, purified from *Bacillus* sp., had an optimum temperature of 40 °C [22], with similar results being reported for an alginate lyase from *Vibrio* sp., a marine bacterium [23].

Figure 4C indicates that AL2 is thermostable between 10 and 37 °C, after which the enzyme activity rapidly decreases, similar to that of Alg2A from *Flavobacterium* sp. S20 [24]. The commercial alginate lyase displayed a wider thermostability range than AL2—activity remained relatively consistent between 10 and 50 °C. *Flammeovirga* rAly5 was thermostable between 10 and 50 °C [22]. The *Flammeovirga* sp., NJ-04, also had a thermostable range close to this [17]. These are similar ranges to that for the commercial enzyme characterised in this study (Figure 4C), indicating that some alginate lyases have very wide thermostability ranges.

The effect of NaCl on the activity of the commercial alginate lyase and AL2 was examined by adding different NaCl concentrations (0–1 M) to 0.1 mg mL^−1^ enzyme in sodium alginate substrate (Cat no. W201502, purchased from Sigma-Aldrich (St. Louis, MO, USA)). AL2 and the commercial enzyme had their highest specific activities at 0.2 M sodium chloride (Figure 4D), which is lower than the salt concentration in seawater, which ranges from 0.55 to 0.6 M. AL2 activity was significantly increased by the addition of 0.2 M NaCl (*p* < 0.05). In contrast, the activity of the commercial alginate lyase was not significantly affected by the addition of 0.2 M NaCl (*p* > 0.05). These results are consistent with reports in the literature. An optimal NaCl concentration of 0.2 M is similar to that for AlgA from *Bacillus* sp. Alg07 [13].

Although its activity was enhanced by ~50% after adding NaCl, AL2 remained active in the absence of NaCl (Figure 4D), indicating that its activity is not NaCl-dependent. This is not true for many alginate lyases—many are only active in the presence of NaCl [13,23,25]. Considering that the enzymes being described were isolated from marine bacteria, it is no surprise that many are NaCl-dependent and that the activities of others improved in the presence of NaCl [8]. A reason for this may be that the NaCl helps to stabilise the enzyme transition state.

AL2 and the commercial alginate lyase remained stable as NaCl concentrations increased above 0.2 M. The activities of other characterised alginate lyases decreased dramatically as NaCl concentrations increased above 0.2 M [13,25]. AlgH from *Marinimicrobium* sp. H1, AL2, and commercial alginate lyase were salt-tolerant, maintaining activity at salt concentrations of 0.5 M and above [26]. Salt tolerance is a useful characteristic for these enzymes, as there is no need for brown algae to be desalted before enzymatic degradation [26], thus reducing upstream processing in industrial applications.

#### 2.3.2. Enzyme Activity

The hydrolysis of alginate by the recombinant and commercial alginate lyases was performed under optimal conditions to determine the maximal specific activity. The specific activity (µmol h^−1^ mg^−1^ protein) of each enzyme is compared in Table 1.

As displayed in Table 1, AL2 exhibited significantly higher specific activity during the bioconversion of alginate into alginate oligosaccharides under optimal reaction conditions (*p* < 0.05). Therefore, AL2 could be used for the efficient enzymatic hydrolysis of alginate. Enzymatic hydrolysis is a preferred method of hydrolysis as it is specific and results in high AOS production yields, occurs rapidly, and has mild reaction conditions, making it safer for the environment [3].

It should be noted that different definitions of enzyme activity units prevent direct comparison of many reported specific activities for alginate lyases. Most studies on alginate lyases determine activity by looking at the increase in absorbance at 235 nm or 540 nm [27]. Since this study looked at the release of reducing sugars by measuring absorbance at 540 nm, only publications determining enzyme activities using the same methods were selected for comparison and converted to per-hour data.

The activity of AL2 was comparable to another recombinant alginate lyase, AlyH1, from *Vibrio furnissii* H1, which had a specific activity of 144 µmol h^−1^ mg^−1^ [12]. rAly1-T185N, also from a *Flammeovirga* sp. (strain MY04), had a specific activity of 173.7 µmol h^−1^ mg^−1^ [14], which is very similar to that of AL2, but slightly higher. A purified alginate lyase from the marine bacterium *Microbulbifer* sp. ALW1 was reported to have a specific activity of 89.4 µmol h^−1^ mg^−1^ [17]. This activity was similar to that found for the commercial alginate lyase, but much lower than that of AL2.

It should also be noted that the elution sample used (elution 2) still contained contaminating proteins. The presence of these proteins increased the total protein concentration determined by the Bradford assay [28], which would decrease the enzyme’s specific activity when dividing by the total protein present in the elution sample (mg protein). Therefore, further protein purification optimisation in future studies may produce an enzyme with an even higher specific activity.

Most alginate lyases show increased activity in the presence of ions, including Mg^2+^ and K^+^ [29], which means that adding these compounds could significantly increase the enzyme activity of AL2. The stability of the commercial alginate lyase in the study was higher than that of the recombinant AL2. It was reported that adding 20% (*w*/*v*) glycerol improved the stability of a recombinant enzyme, Alys1, from the marine bacterium *Tamlana* sp. s12 [29]. Therefore, glycerol could be added in future studies to enhance the stability of the recombinant enzyme under more extreme pH and temperature conditions.

### 2.4. AOS Molecular Weight (Da) Determination

**Figure 5:**
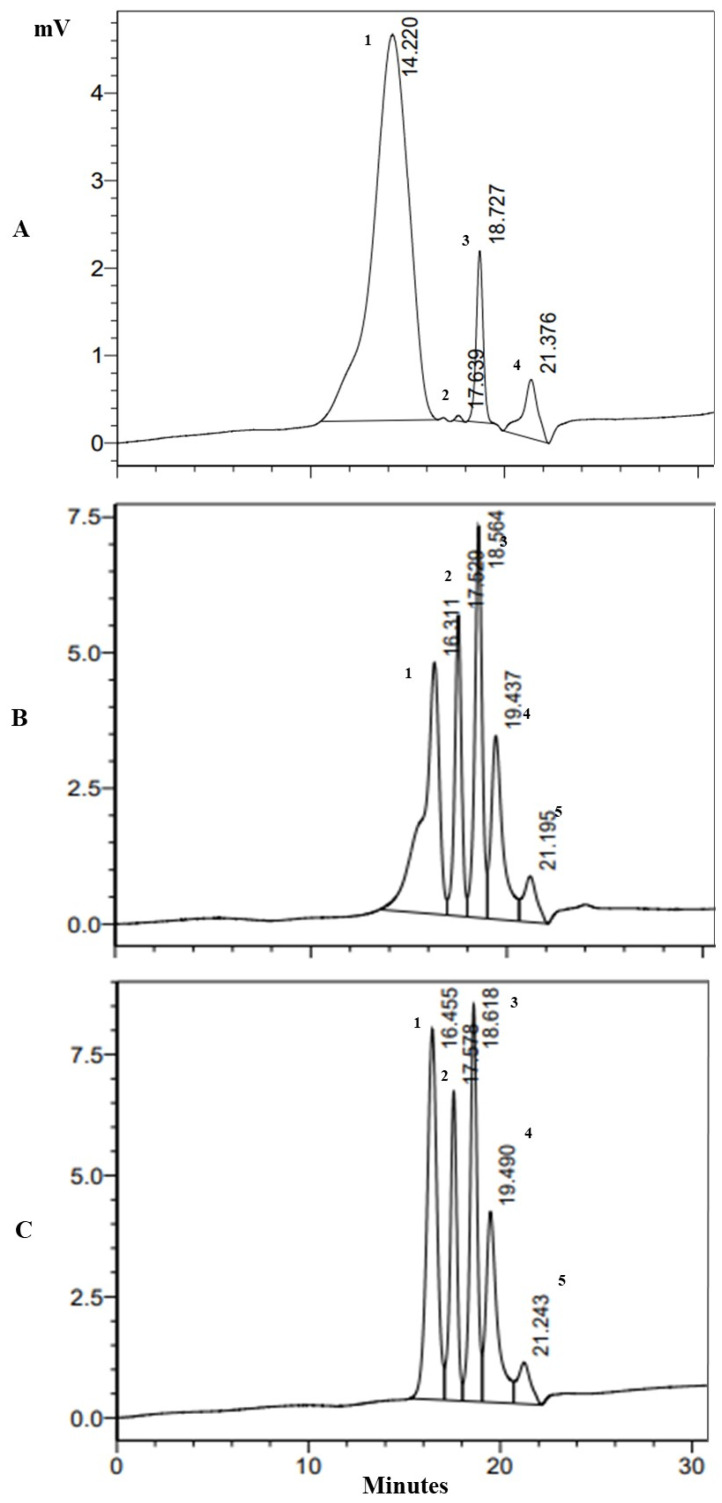
Alginate oligosaccharide molecular weight determination via HP-SEC on a Shodex OHpak SB-806M HQ (8.0 mm × 300 mm) column. (**A**) Unhydrolysed sodium alginate chromatogram (5 mg mL^−1^) (**B**) Alginate oligosaccharide fractions produced from sodium alginate hydrolysis by *Flammeovirga* AL2 (5 mg mL^−1^) (**C**) Alginate oligosaccharide fractions produced from sodium alginate hydrolysis by the commercial alginate lyase (5 mg mL^−1^). Numbers 1–5 represent fragment peak numbers.

The HPLC peaks of the unhydrolysed sodium alginate and the oligosaccharide fragments produced by both the recombinant and commercial alginate lyases were analysed by HP-SEC and are compared in Figure 5. Pullulan standards were used. The sizes (Da) of all the fragments are indicated in Table 2.

Sodium alginate had a high molecular weight of 127 kDa (Table 2). Enzymatic hydrolysis of sodium alginate by AL2 produced several fragments of sizes 5.8, 0.99, 0.22, 0.06 kDa, and 4 Da (Table 2). Sodium alginate hydrolysis by the commercial alginate lyase also yielded several fragments with sizes of 4.78, 0.93, 0.21, 0.06 kDa, and 4 Da (Table 2). The hydrolysis patterns of both alginate lyases were very similar. These are relatively large fragments, indicating that both enzymes are endo-cleaving enzymes. Endo-cleaving enzymes degrade long alginate polymers within the chain, yielding oligosaccharides as their primary products. In contrast, exotype alginate lyases cleave the glycosidic bonds at the terminal residues, producing dimers and monomers as their main products [30].

The AOS fragments produced by commercial alginate lyase and AL2 were much larger than those reported in the literature. For example, the fragments produced by an endo-type alginate lyase from *Bacillus litoralis* had retention times of 82.2, 76.9, and 73.1 min [31]. These times correspond to the production of dimers, trimers, and tetramers, respectively. However, it should be noted that the substrate was alginate from *Sargassum horneri* rather than the commercial sodium alginate used in this study. The HPLC analysis on *Flammeovirga* sp. (strain MY04) hydrolysis was more similar to what has been observed in this study [14].

### 2.5. Inhibition Studies of AOS on Dietary Starch-Degrading Enzymes

Alginate oligosaccharides have shown promising bioactivity compared to sodium alginate, including various anti-diabetic properties. Therefore, the anti-diabetic potential of these oligos was investigated by testing their inhibitory potential against the starch-digesting enzymes α-amylase and α-glucosidase (see Supplementary Material). The crude AOS samples were incubated with these enzymes (see Supplementary Material for methods carried out). Neither the AOS samples nor the unhydrolysed sodium alginate exhibited any inhibitory activity against α-amylase or α-glucosidase (Figure S1).

## 3. Materials and Methods

### 3.1. Database Mining and Identification of Flammeovirga AL2 for Recombinant Expression

The CAZy database (http://www.cazy.org/) was screened for endo-acting alginate lyases which could be applied in alginate degradation studies. The PL7 family was identified as the most promising, specifically the alginate lyases produced by *Flammeovirga* sp. strain MY04. The *algL-5* gene (KT266807.1), used in the study by [22], was selected for further investigation. The predicted protein sequence was submitted to SignalP 6.0, and the predicted signal peptide was removed from the gene sequence prior to synthesis, giving a predicted protein of 540 aa vs. the 566 aa of algL-5. The gene was also codon-optimised for expression in *E. coli*. The truncated, codon-optimised gene was designated *Flammeovirga* AL2. The recombinant plasmid, pET-28a(+), harbouring the truncated, codon-optimised gene encoding for the *Flammeovirga* alginate lyase 2 (AL2), was synthesised by Synbio Technology (Monmouth Junction, NJ, USA). The recombinant plasmid (which also encodes an N-terminal 6x His tag attached to the recombinant enzyme) was transformed into Escherichia coli ArcticExpress (DE3) RP chemically competent cells (Agilent Technologies, Santa Clara, CA, USA). Briefly, 50 ng of plasmid was added to 50 µL of competent cells and incubated on ice for 30 min. The reaction mixture was heat-shocked at 42 °C for 20 s and incubated on ice for 2 min, and 450 µL of SOC (super optimal broth with catabolic repressor; 2% (*w*/*v*) tryptone, 0.5% (*w*/*v*) yeast extract, 10 mM NaCl, 2.5 mM KCl, 10 mM MgCl_2_, 10 mM MgSO_4_, and 20 mM glucose) was added. The transformation reactions were incubated at 37 °C (with shaking at 160 rpm) for 1 h. In duplicate, 100 µL was plated onto Luria agar (1% (*w*/*v*) tryptone, 0.5% (*w*/*v*) yeast extract, and 0.5% (*w*/*v*) NaCl supplemented with 50 µg mL^−1^ kanamycin and 25 µg mL^−1^ gentamycin). Clones were picked and sub-cultured in Luria broth (supplemented with the same antibiotics) and incubated at 37 °C overnight, with shaking at 160 rpm. Glycerol stocks (20% *v*/*v* final glycerol concentration) were prepared and stored at −80 °C until use.

The bacterial constructs (1% (*v*/*v*)) were inoculated in 10 mL 2x YT broth (1.6% (*w*/*v*) tryptone, 1.0% (*w*/*v*) yeast extract, and 0.5% (*w*/*v*) NaCl and supplemented with kanamycin (50 µg mL^−1^). The inoculum was incubated overnight at 30 °C with rapid shaking (180 rpm) and then transferred into 100 mL 2x YT broth until the optical density (Abs_600nm_) reached 0.5–0.6. AL2 expression was induced by adding 1 mM isopropyl β-D-1-thiogalactopyranoside (IPTG) to the culture medium and incubated for 24 h at 30 °C with rapid shaking (180 rpm). Culture samples (1 mL) were collected pre-induction and 2, 4, 6, and 24 h after induction for subsequent analysis of protein expression by loading equal protein concentrations and running a 12% SDS-PAGE.

The cells were collected after 24 h by centrifugation at 10,000× *g* for 10 min at 4 °C. Cell lysis was carried out by adding 10 mL lysis buffer (50 mM Tris, 300 mM NaCl, 0.1% (*w*/*v*) Tween 20), and 10 mg lysozyme per gram of pellet. After a 2 h incubation period at 25 °C with gentle shaking (80 rpm), the lysate was stored at −80 °C.

### 3.2. AL2 Purification

The lysate was thawed on ice and centrifuged at 10,000× *g* for 10 min at 4 °C. The recombinant alginate lyase supernatant was incubated overnight with Ni-NTA resin (HisPur^TM^ Ni-NTA resin, Thermo Fisher Scientific^TM^, Waltham, MA, USA) at 4 °C with gentle shaking (80 rpm). The protein-bound Ni-NTA resin was first washed using two-bed volumes of lysis buffer (50 mM Tris, 300 mM NaCl, and 0.1% (*w*/*v*) Tween 20). The first wash was followed by a second two-bed wash using wash buffer (50 mM Tris, 300 mM NaCl, and 10 mM imidazole, pH 7.4) to remove weakly bound proteins from the column. The target enzyme was then eluted from the column using elution buffer 1 (50 mM Tris, 300 mM NaCl, and 200 mM imidazole, pH 7.4), followed by a second elution using elution buffer 2 (50 mM Tris and 500 mM imidazole, pH 8). Samples were collected from the column after each wash and elution step, and the protein purification process was analysed using 12% SDS-PAGE. The molecular weight of the purified enzyme was determined using a protein ladder (Bio-Rad Precision Plus Protein^TM^ Standards Dual Colour). The protein concentration of the purified enzyme was determined by the Bradford assay [28] using a standard curve constructed with bovine serum albumin (BSA).

### 3.3. Alginate Lyase Characterisation

#### 3.3.1. Optimum pH

The optimal pH of the enzyme was determined by incubating the enzyme (1% *v*/*v*) in sodium alginate substrate (10 mg mL^−1^) dissolved in Britton–Robinson universal buffer (0.1 M acetic acid, 0.1 M boric acid, 0.1 M phosphoric acid) in a pH range of 4–10 (in increments of 1 pH unit) at 37 °C for 12 h with shaking at 130 rpm. The enzyme activity was measured using the 3,5-dinitrosalicylic (DNS) acid method [27]. The absorbance was read at 540 nm using a spectrophotometer (Epoch™2 Microplate Spectrophotometer, BioTek, Winooski, VT, USA). The same protocol was repeated for the commercial alginate lyase (0.1 mg mL^−1^) as a positive control.

#### 3.3.2. Optimum Temperature and Thermostability

The optimum temperature of the enzyme was determined by incubating the enzyme in sodium alginate (10 mg mL^−1^) dissolved in Tris-HCl buffer (50 mM Tris, 100 mM NaCl, pH 9) over a range of reaction temperatures (12–60 °C) at increments of 10 °C. Thermal stability was determined by preincubating the enzyme in Tris-HCl buffer (50 mM Tris, 100 mM NaCl, pH 9) over a range of temperatures (10–60 °C) for 1 h, followed by an incubation period of 12 h in sodium alginate substrate with shaking at 130 rpm. The same protocol was repeated for the commercial alginate lyase, which served as a positive control, but at pH 8.0. Enzyme activity was determined using the DNS acid method [27].

#### 3.3.3. Effect of Sodium Chloride

The effect of sodium chloride on the activity of AL2 and the commercial alginate lyase was investigated by incubating the enzymes in sodium alginate substrate (10 mg mL^−1^) dissolved in Tris-HCl buffer (Tris 50 mM) at a range of NaCl concentrations (from 0–1 M, increasing in intervals of 0.2 M). An additional concentration of 0.55 M was included, as it is the lowest concentration of sodium chloride that marine saltwater is reported to have. The reaction was carried out at pH 9.0 for AL2 and pH 8.0 for the commercial alginate. The enzymes were incubated with the substrate–NaCl mix for 12 h at 37 °C, with shaking at 130 rpm. Enzyme activity was determined using the DNS acid method [27].

#### 3.3.4. Enzyme Activity

The DNS method [27] was used to determine enzyme activity. Sodium alginate (10 mg mL^−1^), purified *Flammeovirga* AL2 (20 µL), or commercial alginate lyase (0.1 mg mL^−1^), as well as Tris-HCl buffer (50 mM Tris, 100 mM NaCl), were combined. A substrate control replacing the enzyme with buffer was included. The enzymatic reactions were incubated at the optimal temperature and pH for 12 h, with shaking at 130 rpm. A D-glucose standard curve was generated to determine the enzyme’s specific activity (µmol h^−1^ mg^−1^ protein).

### 3.4. Alginate Oligosaccharide High-Performance Size Exclusion Chromatography (HP-SEC)

Sodium alginate (10 mg mL^−1^) dissolved in Tris-HCl buffer (50 mM Tris, 100 mM NaCl) was hydrolysed by AL2 (1% *v*/*v*) and the commercial enzyme (0.1 mg mL^−1^) for 24 h. The sample was freeze-dried and resuspended in 0.1 M NaNO_3_ mobile phase to a final concentration of 5 mg mL^−1^ and filtered through a 0.22 µm nylon membrane (Membrane Solutions, Auburn, WA, USA). The same procedure was repeated for sodium alginate (5 mg mL^−1^). The hydrolysates and sodium alginate were analysed by Size Exclusion High-Performance Liquid Chromatography (referred to as HP-SEC) to determine the molecular weights of the AOS and the unhydrolysed polysaccharide. An 8 mm × 300 mm Shodex OHpak SB-806M HQ column (Showa Denko, Tokyo, Japan) was used, following the manufacturer’s recommendations. A high-performance liquid chromatography refractive index detector (HPLC-RID) was used, and the column temperature was set at 30 °C. A 0.1 M NaNO_3_ (aq) solution was used as the mobile phase at a flow rate of 0.5 mL min^−1^. The samples were then injected into the column at a volume of 10 µL. A sodium alginate standard curve was constructed using pullulan standards (Shodex, Tokyo, Japan)), and this was used to determine the molecular weights of the AOS fragments and the unhydrolysed sodium alginate used in the study.

### 3.5. Statistical Analysis

Data are represented as triplicates for all experiments (unless otherwise stated) and expressed as means ± standard deviations (SD). Statistical differences between data were assessed using *t*-tests. The data were considered significantly different when the *p*-value was less than 0.05 (*p* < 0.05). The *t*-tests were performed using GraphPad Prism software version 10 (GraphPad Inc., Boston, MA, USA).

## 4. Conclusions

This study successfully expressed and purified a truncated, codon-optimised, recombinant alginate lyase (AL2) from the marine bacterial genus *Flammeovirga.* Our results showed that AL2 has significantly higher hydrolytic activity than the commercial alginate lyase. The alginate lyases successfully produced AOS at high concentrations. AOS fragments have been shown to display various anti-diabetic properties, including the inhibition of starch-degrading enzymes, such as α-glucosidase and α-amylase. Therefore, the potential of these fragments as starch-degrading enzyme inhibitors was investigated. AOS fragments did not inhibit these enzymes in our study, so further studies in this regard are suggested. Importantly, this study does highlight that AL2 may be a potentially useful industrial enzyme for the hydrolysis of alginate into AOS.

## Figures and Tables

**Table 1:** Enzyme-specific activities under optimal reaction conditions ^1^.

Enzyme	Specific Activity (µmol h^−1^ mg^−1^ protein)
AL2	151.6 ± 12.8
Commercial alginate lyase	96.9 ± 13.1
*p*-value	0.001

^1^ The specific activity (µmol h^−1^ mg^−1^ protein) of AL2 was determined at pH 9 after a 12 h incubation period at 37 °C. The commercial alginate lyase’s specific activity (µmol h^−1^ mg^−1^ protein) was determined at pH 8 after a 12 h incubation period at 37 °C. Data points represent mean values ± SD (n = 4). The *p*-value indicates that these specific activities are significantly different.

**Table 2:** A comparison of alginate and alginate oligosaccharide fragment molecular weights (Da) ^1^.

	Fragment				
	1	2	3	4	5
Sodium Alginate	127,659 ± 6579	841	175 ± 4	4 ± 0.2	-
AL2 AOS	5834 ± 6	993 ± 8	222 ± 6	62 ± 0.6	4 ± 0.4
Commercial AL AOS	4775 ± 69	931 ± 17	206 ± 6	58 ± 2	4 ± 0.3

^1.^ Sodium alginate represents the pre-enzymatic hydrolysis sample. AL2 AOS and the commercial alginate lyase (AL) AOS are post-enzymatic hydrolysis samples. The data values represent mean values ± SD (n = 2).

## Data Availability

The data presented in this study are available on request from the corresponding author.

## Supplementary Materials

The following supporting information can be downloaded at: https://www.mdpi.com/article/10.3390/molecules29235578/s1, Figure S1: Inhibitory effects of alginate and alginate oligosaccharide fractions on starch-digesting enzymes.
