# Supplementary material for: Expression, Purification, and Characterisation of Recombinant Alginate Lyase (Flammeovirga AL2) for the Bioconversion of Alginate into Alginate Oligosaccharides
Source: Molecules. 2024 Nov 26;29(23):5578. doi: 10.3390/molecules29235578 (PMC11643991; doi:10.3390/molecules29235578)
Supplement: Supplementary file 1 [file molecules-29-05578-s001.zip › molecules-3300317-supplementary.pdf]

Supplementary Material:

Results:

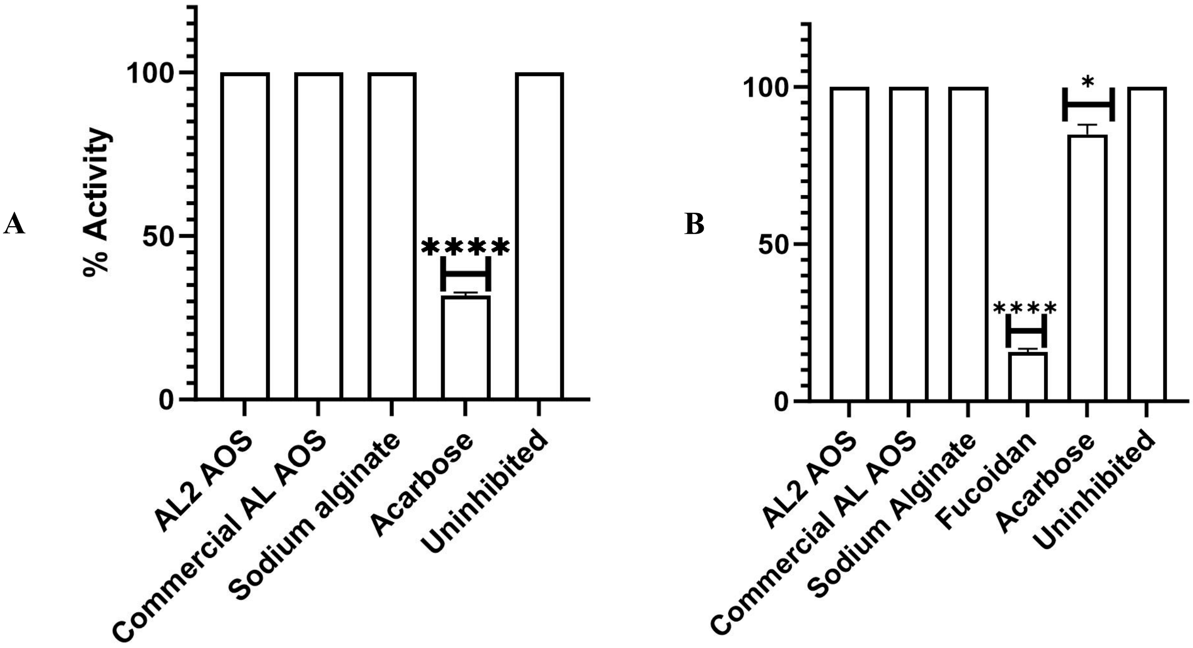

Figure S1: Inhibitory effects of alginate and alginate oligosaccharide fractions on starch-digesting enzymes (**A**) α-amylase inhibition. The % activity represents the remaining activity displayed by α-amylase. Acarbose is included as a positive control. Data points represent mean values ± SD (n=3). **** denotes the significant difference (p < 0.001) from the uninhibited reaction. (**B**) α-glucosidase inhibition. The % activity represents the remaining activity displayed by α-glucosidase. Fucoidan from *Fucus vesiculosus* and acarbose are included as positive controls. Data points represent mean values ± SD (n=3). **** denotes the significant difference (p < 0.001) from the uninhibited reaction. * denotes the significant difference (p < 0.05) from the uninhibited reaction.

Methods and Materials:

Enzyme inhibition studies

α-amylase inhibition assay

The release of D-glucose was measured to determine the inhibitory potential of *Flammeovirga* AL2 and commercial alginate lyase hydrolysates (1 mg.ml^{-1}) and unhydrolysed sodium alginate (1 mg.ml^{-1}) against α-amylase (0.3 mg.ml^{-1}). The enzyme was incubated with the inhibitors at 37°C for 45 minutes in a 2% (w/v) potato starch substrate in phosphate buffer (50 mM monosodium dihydrogen phosphate, 50 mM disodium hydrogen phosphate, pH 7.0) at a total reaction volume of 400 µL. Acarbose (1 mg.ml^{-1}), a known α-amylase inhibitor, was used as a positive control. A substrate control was prepared by replacing the inhibitor with buffer. An uninhibited control was included to determine the remaining activity (%) in the presence and absence of inhibitors. The reaction was terminated by boiling at 100°C and centrifugation at 13000 *g* for 5 minutes. The supernatant was incubated with GOPOD reagent at 40°C for 20 minutes. The absorbance was then read at 510 nm (Epoch™2 Microplate Spectrophotometer, BioTek).

Enzyme inhibition (remaining activity) % =

$$100 - \frac{(glucose\ released\ by\ control - glucose\ released\ by\ test\ reaction)}{glucose\ released\ by\ control}\ x\ 100$$

α-glucosidase inhibition assay

The potential of *Flammeovirga* AL2 and commercial alginate lyase hydrolysates (1 $mg.ml^{-1}$), and unhydrolysed sodium alginate (1 $mg.ml^{-1}$) as α-glucosidase (0.01 $mg.ml^{-1}$) inhibitors was investigated by observing the release of p-nitrophenol in the presence of these compounds. Fucoidan from *Fucus vesiculosus* (1 $mg.ml^{-1}$), a potent α-glucosidase inhibitor, and acarbose (1 $mg.ml^{-1}$) were used as positive controls. A final concentration of 0.4 mM pNPG substrate was combined in phosphate buffer (50 mM monosodium dihydrogen phosphate and 50 mM disodium hydrogen phosphate, pH 7.0). The reaction was allowed to proceed for 20 minutes at 37°C, after which it was terminated using 2 M sodium carbonate. An uninhibited control was included to determine the remaining activity (%) in the presence and absence of inhibitors. The absorbance was measured at 405 nm (Epoch™2 Microplate Spectrophotometer, BioTek) to determine the amount of p-nitrophenol released.

Enzyme inhibition (remaining activity) % =

$$100 - \frac{(p\ nitrophenol\ released\ by\ control - p\ nitrophenol\ released\ by\ test\ reaction)}{p\ nitrophenol\ released\ by\ control}\ x\ 100$$

<u>Discussion:</u>

The AOS fragments produced in this study did not have an inhibitory effect on α-amylase or α-glucosidase (Fig. S1) and, therefore, displayed no anti-diabetic properties. Considering that the fragments present in the highest concentration had high molecular weights, it is likely that they were still too large to interact with the starch-degrading enzymes. Alternatively, the smaller fragments may have been too small. According to Liu *et al*. (2019), AOS fragments with low molecular weights have shown good bioactivity (Wang *et al.,* 2021). AOS fragments with molecular weights of ~3000 Da have been reported to stimulate insulin secretion *in vitro* (Xing *et al.,* 2020). As mentioned, the composition of AOS also affects their bioactivity. Therefore, the composition of the AOS fragments used in this study may have resulted in their lack of inhibition towards the starch-degrading enzymes. To the best of our knowledge, no other study in the literature has investigated the impact of AOS in inhibiting the amylolytic enzymes as we have done in this study. Therefore, no comparisons to other reports in the literature are possible.
